# The complete mitochondrial genome of the cat flea, *Ctenocephalides felis*

**DOI:** 10.1080/23802359.2020.1823259

**Published:** 2020-10-05

**Authors:** Victoria I. Verhoeve, Mariah L Plumer, Timothy P. Driscoll, Kevin R. Macaluso, Abdu F. Azad, Joseph J. Gillespie

**Affiliations:** a Department of Microbiology and Immunology, University of Maryland School of Medicine, Baltimore, MD, USA; b Department of Biology, West Virginia University, Morgantown, WV, USA; c Department of Microbiology and Immunology, College of Medicine, University of South Alabama, Mobile, AL, USA

**Keywords:** Cat flea, *Ctenocephalides felis*, Siphonaptera, mitochondria, genome, PacBio sequencing

## Abstract

The cat flea, *Ctenocephalides fells*, is widely recognized as a global veterinary pest and a vector of pathogenic bacteria. We recently reported on the *C*. *felis* nuclear genome, which is characterized by over 38% protein coding gene duplication, extensive tRNA gene family expansion, and remarkable gene copy number variation (CNV) between individual fleas. Herein, we describe the assembly of the *C*. *felis* mitochondrial genome, a novel resource for comparative genomics of fleas and other insects. The order and content of mitochondrial genes is highly consistent with four previously sequenced flea mitochondrial genomes, limiting CNV to siphonapteran nuclear genomes.

With over 2,500 identified species across the globe, fleas are notorious veterinary pests and vectors of pathogens, including *Rickettsia typhi* (murine typhus), *R*. *felis* (murine typhus-like illness), *Bartonella henselae* (cat-scratch disease), and myxoma virus (Myxomatosis) (Bertagnoli and Marchandeau 2015; McElroy et al. 2010; Mullen and Durden 2002). Speciation of fleas is reliant on distinguishing morphological features; however, studies have also used certain mitochondrial genes for systematic analyses (Lawrence et al. 2019; McKern et al. 2008; Whiting et al. 2008). To date, four full mitochondrial genomes have been sequenced in the order Siphonaptera, representing a potential untapped source of genomic variation for clearer evolutionary inferences (Cameron 2015; Hystrichopsylla 2019; Tan et al. 2018; Xiang et al. 2017). We assembled the mitochondria genome of the cat flea, *Ctenocephalides felis,* using reads generated from our sequencing of the cat flea nuclear genome (Driscoll et al. 2020). While the cat flea nuclear genome exhibits unprecedented genome plasticity evinced by excessive gene duplication, the mitochondrial genome of the cat flea is consistent with other Siphonaptera mitochondrial genomes sequenced to date and contains no evidence of genome rearrangements or duplications.

To sequence the genome of the cat flea, unfed female *C. felis* (*n* = 250) from the Elward Laboratory colony (Soquel, CA, USA) were obtained in January 2018 and pooled for high molecular weight DNA extraction followed by long-read sequencing on the PacBio Sequel. The sample DNA was deposited in the arthropod repository at the University of Maryland Baltimore under accession Cf102787-2018.

Corrected PacBio reads were assembled with Canu (version 1.5) (Koren et al. 2017) in ‘pacbio-raw’ mode; the expected mitochondrial genome was compiled into a single 20,873 bp contig at 2267x coverage. Even with high coverage, the mitogenome could not be circularized informatically or with PCR techniques due to two distinct, contiguous AT-rich repeat regions spanning nearly 5000 bases combined. The mitochondrial genome was preliminarily annotated with a combination of MITOS (using the invertebrate genetic code with default parameters) (Bernt et al. 2013) and GeSeq (with default parameters and flea mitochondrial genomes as reference sequences) (Tillich et al. 2017). The complete flea mitochondrial genomes included in the GeSeq analyses were *Jellisonia amadoi* (NC_022710.1)*, Ceratophyllus wui* (MG886872.1)*, Dorcadi ioffi* (MF124314.1) *and Hystrichopsylla weida qinlingensis* (MH259703.1). Both MITOS and GeSeq did not predict complete open reading frames for any protein coding genes, resulting in truncated gene predictions. After annotation, the Canu-assembled mitochondrial genome contained multiple split genes in six protein coding genes requiring further investigation. To supplement the preliminary annotation, open reading frame analyses and BlastN were used to identify full open reading frames for protein coding genes extending in most cases in both 5′ and 3′ directions. Manual sequence analysis revealed split genes that appeared to be missing bases within homopolymer stretches resulting in truncated open reading frames. Targeted PCR amplification and Sanger sequencing resolved deletions at sites often containing stretches of four or more A or T. Additionally, paired-end 250 basepair read Illumina sequencing of *C. felis* from the same Elward Laboratory colony resolved the remaining deletions after examining read pileups for evidence of additional encoded bases.

The *C. felis* mitochondrial genome (Genbank accession number: MT594468) encodes the full repertoire of 37 genes, including 22 tRNAs, 13 protein coding genes, and 2 rRNAs with the conserved synteny observed in other Siphonaptera mitogenomes and the general insect mitochondrial gene order (Cameron 2015). The major strand is composed of 83.1% A + T and all protein coding genes are similar in size to homologs in other fleas with no evidence of gene truncations or major rearrangements. There are few non-coding positions within the conserved block of encoded genes. The singular intergenic spacer encoded is 50 bases in length and occurs between *trnS2* and *nad1*. The cat flea mitogenome is at least 2000 bases greater in length than *Ceratophyllus wui*, the next largest sequenced flea mitogenome (Tan et al. 2018). The increase in the length of the *C. felis* mitochondrial genome is due to the longer AT-rich repeat regions flanking the core 37 gene segment, not due to additional internal spacer regions. All protein coding genes begin with canonical start codons (ATN) with the exception of *atp8* starting with a TTG start codon. Stop codons are equally split between full TAA stop codons and incomplete T stop codons with the exception of *ctyb*, which has a TAG stop codon.

Phylogeny estimation of full proteomes from the five siphonapteran mitochondrial genomes corroborates previously determined flea relationships (Whiting et al. 2008), barring the lack of complete mitochondrial genome sequences for most of the siphonapteran families (Figure 1). In contrast to the extreme genome size variation reported for *C*. *felis*, as well as the rat flea *Xenopsylla cheopis* (Driscoll et al. 2020), the observed genome stasis and phylogenetic utility of flea mitochondrial genomes implicates these resources as prudent tools for future analyses on flea systematics and epidemiology of flea-borne diseases.

**Figure 1.**
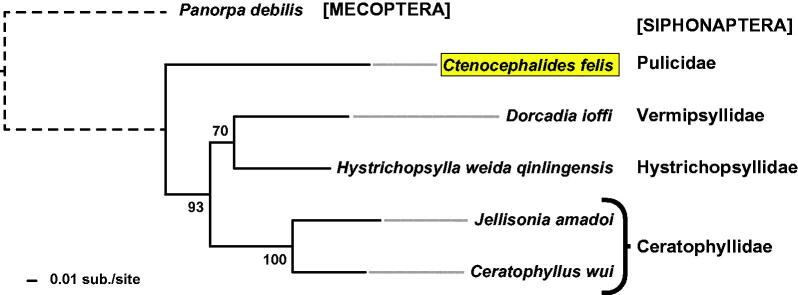
Phylogeny estimation based on five siphonapteran proteomes. Protein sequences (n = 12: ATP synthase F0 subunits 6 & 8, cytochrome b, cytochrome c oxidase subunits I & III, and NADH dehydrogenase subunits 1-6 & 4 L) from 6 sequenced siphonapteran mitogenomes (*Ctenocephalides felis*, *Jellisonia amadoi*, *Ceratophyllus wui*, *Dorcadia ioffi*, and *Hystrichopsylla weida qinlingensis*) plus *Panorpa debilis* (Mecopteran) were independently aligned with MUSCLE v3.8.31 using default parameters. Aligned sequences were concatenated into a single data set (3,505 positions) for phylogeny estimation using RAxML v8.2.4, under the gamma model of rate heterogeneity, WAG model of substitution, and estimation of the proportion of invariant sites. Branch support was assessed with 500 pseudo-replications. Final ML optimization likelihood was -26546.055122.

## Data Availability

The data for this study are openly available in GenBank at https://www.ncbi.nlm.nih.gov/nucleotide/ under accession number MT594468.
